# Osteogenesis‐Inducing Chemical Cues Enhance the Mechanosensitivity of Human Mesenchymal Stem Cells for Osteogenic Differentiation on a Microtopographically Patterned Surface

**DOI:** 10.1002/advs.202200053

**Published:** 2022-04-04

**Authors:** Jianxiang He, Dongqi You, Qi Li, Jiabao Wang, Sijia Ding, Xiaotong He, Haiyan Zheng, Zhenkai Ji, Xia Wang, Xin Ye, Chao Liu, Hanyue Kang, Xiuzhen Xu, Xiaobin Xu, Huiming Wang, Mengfei Yu

**Affiliations:** ^1^ Key Laboratory of Oral Biomedical Research of Zhejiang Province Stomatology Hospital School of Stomatology Zhejiang University School of Medicine Zhejiang Provincial Clinical Research Center for Oral Diseases Hangzhou 310006 P. R. China; ^2^ School of Materials Science and Engineering and Institute for Advanced Study Tongji University Shanghai 201804 P. R. China; ^3^ School of Stomatology The First Affiliated Hospital of Zhejiang University School of Medicine Hangzhou 310003 P. R. China

**Keywords:** chemical cues, hollow‐micro‐frustum‐arrays, mechanosensitivity, mesenchymal stem cells, osteogenic differentiation

## Abstract

Mechanical cues are widely used for regulating cell behavior because of their overarching, extensive, and non‐invasive advantages. However, unlike chemical cues, mechanical cues are not efficient enough to determine cell fate independently and improving the mechanosensitivity of cells is rather challenging. In this study, the combined effect of chemical and mechanical cues on the osteogenic differentiation of human mesenchymal stem cells is examined. These results show that chemical cues such as the presence of an osteogenic medium, induce cells to secrete more collagen, and induce integrin for recruiting focal adhesion proteins that mature and cascade a series of events with the help of the mechanical force of the scaffold material. High‐resolution, highly ordered hollow‐micro‐frustum‐arrays using double‐layer lithography, combined with modified methacrylate gelatin loaded with pre‐defined soluble chemicals to provide both chemical and mechanical cues to cells. This approach ultimately facilitates the achievement of cellular osteodifferentiation and enhances bone repair efficiency in a model of femoral fracture in vivo in mice. Moreover, the results also reveal these pivotal roles of Integrin α2/Focal adhesion kinase/Ras homolog gene family member A/Large Tumor Suppressor 1/Yes‐associated protein in human mesenchymal stem cells osteogenic differentiation both in vitro and in vivo. Overall, these results show that chemical cues enhance the microtopographical sensitivity of cells.

## Graphical abstract

Chemical cues enhance the mechanical sensitivity of mesenchymal stem cells through Integrin α2/Focal adhesion kinase/Ras homolog gene family member A/Large Tumor Suppressor 1/Yes‐associated protein pathway. Further, double‐layer lithography and anisotropic etching is used to fabricate high‐resolution, highly ordered 3D hollow‐micro‐frustum‐arrays, combined with modified methacrylate gelatin loaded with pre‐defined soluble chemicals to provide both chemical and mechanical cues to cells to achieve osteogenic differentiation.

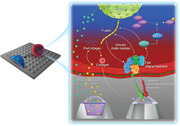

## 1 Introduction

From embryonic development to generation of adult tissues, stem cells and their local microenvironment communicate through chemo‐mechanical cues to regulate cell fate and to direct developmental processes.^[^ ^1^, ^2^, ^3^ ^]^ During early embryonic development, non‐muscle myosin II provides intrinsic mechanical stretching and compression forces to regulate embryonic morphogenesis.^[^ ^4^ ^]^ The embryo also receives external mechanical inputs from the surrounding fluid,^[^ ^5^ ^]^ along with the morphogens sonic hedgehog (SHH) and retinoic acid secreted in the blastocyst, which determine embryonic patterning.^[^ ^6^ ^]^ As development proceeds, cell‐extracellular matrix (ECM) interactions occur more frequently than cell–cell interactions, and progenitor cell differentiation begins.^[^ ^7^ ^]^ The mechanical properties of the ECM provide a template for organ growth, and the chemical properties guide the direction of organ growth. These two processes work synergistically to form highly organized developmental patterns.^[^ ^8^ ^]^

Materials mimicking the properties of natural ECM provide physiologically relevant cellular microenvironments to achieve unique biological activities. In vitro mechanical forces can shape cells and signal them to regulate cellular metabolism.^[^ ^9^, ^10^, ^11^ ^]^ Mechanical signaling plays a primary role in the cytoskeleton, polarity, endocytosis, nuclear structure, and organelle functions. This suggests that every aspect of cellular behavior is influenced by cellular mechanical transduction.^[^ ^12^, ^13^, ^14^ ^]^ Further, the materials used to provide mechanical cues are highly stable and difficult to dissolve in vivo, which help prevent invasive contamination.^[^ ^15^ ^]^ Although mechanical cues are widely used at the cellular level, the effect of mechanical forces on cell fate is unremarkable compared to that of soluble chemical–biological factors.^[^ ^16^, ^17^, ^18^, ^19^ ^]^ Therefore, it is challenging to enhance the effect of physical cues on cells, that is, the mechanical sensitivity of cells.

We found that chemical cues could enhance the microtopographical sensitivity of cells. However, examination of cell or organ development is complicated because there are multiple ways by which chemo‐mechanical cues and interactions affect cells.^[^ ^20^ ^]^ Furthermore, the composition of decellularized tissues and natural ECM may include multiple chemical and mechanical cues, which are often unclear,^[^ ^21^ ^]^ making it difficult to test specific hypotheses. Therefore, studies on the combined effects of chemical‐mechanical cues are lacking.

In this study, we aim to examine the combined effect of chemical and mechanical cues on the osteogenic differentiation of human mesenchymal stem cells. To this end, we first examined the cellular responses to chemical cues with respect to the expression of Yes‐associated protein (YAP), and the subsequent production of collagen and integrin to recruit focal adhesion proteins, which mature and cascade a series of events through mechanical forces, as well as the resulting osteogenesis‐related gene expression changes. Based on this mechanism, we fabricated a biofilm using hollow‐micro‐frustum‐arrays (HMFA) and modified methacrylate gelatin (loaded with pre‐defined soluble chemokines) to provide both chemical and mechanical cues to the cells. We also fabricated the new biofilm to provide chemical and mechanical cues to the cells and achieve cellular osteodifferentiation and enhanced bone repair efficiency in vivo in mice.

## 2 Results

### 2.1 Microtopographical Stimulation Following OM Induction more Efficiently Differentiates Human Mesenchymal Stem Cells (hMSCs) toward the Osteogenic Lineage

**Figure 1:**
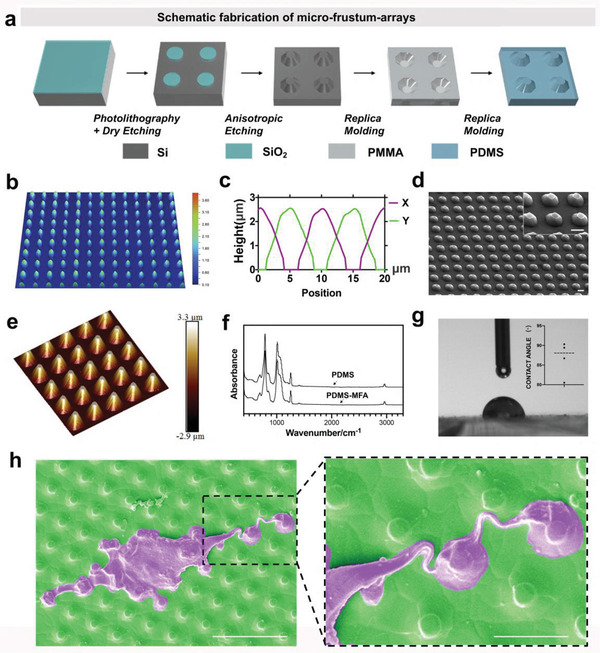
Fabrication process and characterization of PDMS‐based micro‐frustum‐arrays (MFAs). a) Fabrication process of MFAs. b) Confocal laser scanning microscopic image of MFAs. c) The height and diameter of MFAs microparticles were measured in the *X*‐axis and *Y*‐axis directions using confocal laser scanning microscopy. d) Scanning electron microscopic image of MFAs. Scale bar, 5 µm. e) Atomic force microscopic image of MFAs. f) Fourier transform infrared spectroscopy of PDMS and PDMS‐MFA. g) Contact angle analysis of MFAs. h) Scanning electron microscopic image of cells. Cells and surface topography were stained with purple and green, respectively. Scale bar, left 30 µm, right 10 µm.

To evaluate the effect of mechanical cues on cell behavior, we used micro‐frustum‐arrays (MFAs). The fabrication process of polydimethylsiloxane (PDMS)‐based MFA is shown in **Figure** **1**a. First, an oxidized Si (100) substrate with a thin layer of SiO_2_ (100 nm in thickness) was spin‐coated with a layer of a negative photoresist. Next, photolithography was applied to generate micro‐disk arrays of photoresist using a Cr‐photomask with a hollow circular array pattern on it (diameter: 5 µm; pitch: 10 µm). Dry‐etching through a reactive ion etching system was applied to transfer the micro‐disk‐array pattern to the SiO_2_ film underneath, followed by removal of the photoresist. Then, SiO2 micro‐disk arrays were used as a resist in the anisotropic etching of Si (100) (30% KOH solution) to generate Si MFAs. Heat‐assisted imprinting was used to replicate the negative pattern of the MFAs on a PMMA substrate. Finally, a second replica molding process was used to generate the PDMS‐based MFAs. The roughness of the MFA surface was characterized by confocal laser scanning microscopy (CLSM) using the root mean square (RMS) roughness *R*_q_. The MFA had a roughness of ≈525 nm (Figure 1b). The height (≈2.54 µm) and diameter (≈6.42 µm) of MFA microparticles were measured in the *X*‐axis and *Y*‐axis directions using CLSM (Figure 1c). Scanning electron microscopy (SEM) and atomic force microscopy (AFM) images demonstrated the uniformity and orderliness of the MFA microparticles (Figure 1d,e; Figure S1a, Supporting Information). The Fourier transform infrared (FTIR) spectra of PDMS‐MFA is similar to that of PDMS (Figure 1f).^[^ ^22^ ^]^ Characteristic vibrations of siloxane chains are strong IR bands observed at 1010 cm^−1^ (asymmetric Si—O—Si stretching). Other characteristic IR signatures of PDMS are related to C—Si—C groups: 790 cm^−1^ (asymmetric stretching), 700 cm^−1^ (symmetric stretching). Methyl groups have characteristic bands at 2962 cm^−1^ (stretching), 1260 cm^−1^ (deformation), and 694 cm^−1^ (rocking). The contact angle of the MFAs was 86.7 ± 6.22°. To confirm that the MFA substrate supports the adherence and culture of human mesenchymal stem cells (hMSCs) without compromising their viability, hMSCs were inoculated on the MFAs. SEM images and biocompatibility studies showed efficient adhesion and viability of hMSCs after 5 days of culture (Figure 1e; Figure S1b,c, Supporting Information), confirming the good cytocompatibility of the MFA. Interestingly, hMSCs attach better to the top of the microparticles, which aided in obtaining the subsequent proof of mechanism and chemical factor delivery.

**Scheme 1:**
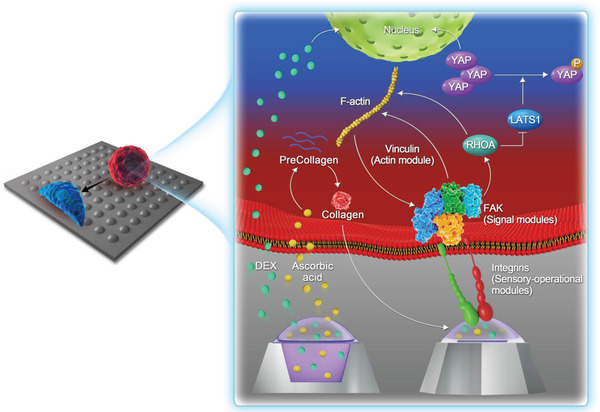
Schematic illustration of the postulated mechanism. Osteogenic medium (OM) promotes the expression of YAP and RUNX2, and the YAP expression is accompanied by compensatory phosphorylation. The micro‐frustum‐array (MFAs) inhibits YAP phosphorylation through mechanical signals including INTA2/FAK/RHOA/LATS1, which are triggered by COL‐I secretion is induced by ascorbic acid, a component of the OM.

Precise and efficient induction of hMSC differentiation only toward the osteogenic lineage, rather than to the connective tissue, is crucial for successful bone regeneration. To investigate the relationship between MFA and hMSC fate, we seeded hMSCs on MFA substrates and then analyzed the expression of osteogenic differentiation markers and transcription factors such as Collagen I (COLI), Osteopontin (OPN), and RUNX Family Transcription Factor 2 (RUNX2) at the protein and mRNA levels. All three osteogenic genes were upregulated in hMSCs cultured on MFA substrates with a growth medium (Figure S1d–g, Supporting Information). However, the effect of MFAs on osteogenesis was not as obvious as that of the osteogenic medium (OM) (Figure S1h–k, Supporting Information).

**Figure 2:**
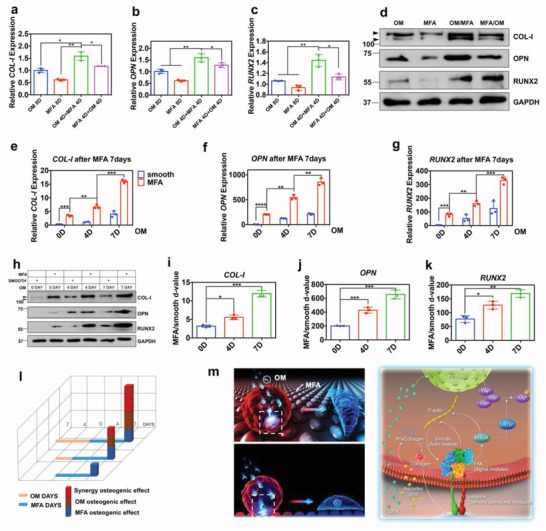
Osteogenic medium (OM) induction, followed by microtopographic stimulation induces hMSC differentiation toward the osteogenic lineage more efficiently. a–c) qPCR and d) western blot analyses of osteogenic genes and their protein expression during different culture conditions. The hMSCs were first induced using OM and then seeded on MFA substrates to show better osteogenesis ability. OM represents hMSCs cultured in OM on a smooth substrate for 8 days; MFA represents hMSCs cultured in the growth medium on MFA for 8 days; OM/MFA represents hMSCs cultured in OM for the first 4 days and then on MFA for the next 4 days; MFA/OM represents hMSCs cultured on MFA for the first 4 days and then in OM for the next 4 days. All data are presented as the mean ± SD, *p* values are based on Student's *t* test, *n* = 3 per group, *p < 0.05, **p < 0.01. e–h) Schematics showing that after stimulation by OM, MFA could stably promote the osteogenic differentiation process of hMSCs and the longer OM was used, the better osteogenic differentiation was observed at the gene and protein levels detected through qPCR (e–g) and western blot (h) analyses. All data are presented as the mean ± SD, *p* values are based on Student's *t* test, *n* = 3 per group, *p < 0.05, **p < 0.01, ***p < 0.001, ****p < 0.0001. i–k) *D*‐values of relative gene expression between hMSCs on the MFA and the smooth substrate represent the osteogenic effect of MFA on competent hMSCs. Competent hMSCs showed greater substrate‐sensitivity. The data are presented as the mean ± SD; *p* values are based on Student's *t* test, *n* = 3 per group. *p < 0.05, ***p < 0.001. l) The schematic diagram shows that hMSCs were first stimulated by OM for 0, 4, and 7 days (competent hMSCs); these competent cells were then cultured on MFA substrates or smooth plates for 7 days before collection and analyses. m) The schematic diagram shows the synergistic effect between OM and MFA, which could effectively promote the osteogenic differentiation of hMSCs.

Interestingly, hMSCs that were first induced with OM and then seeded onto MFA substrates showed the best osteogenic ability compared to the cells that were stimulated using OM or MFA (**Figure** **2**a–d). Furthermore, the longer the culture, better osteogenic differentiation and increased osteogenic benefit were obtained with longer cultures in the combination group compared to those with OM alone (Figure 2e–h). In other words, hMSCs induced with OM remained in a competent state with better mechanosensitivity, resulting in a greater osteogenic benefit on MFA substrates. Further, the *d*‐values between the MFA group and smooth substrate group indicated that the efforts generated from the cooperation of chemical and mechanical factors were much higher (≈3.5‐fold for 7 days) compared to a simple summation (Figure 2i–k). For instance, in Figure 2i, the blue column represents the osteogenic effect of MFA for 7 days; the green column represents the total effect minus the effect of OM for 7 days, which was much higher than that of the blue column. Overall, we found that OM induced the mechanical sensitivity of hMSCs and that OM and MFA produced a synergistic effect on the osteogenic differentiation of hMSCs (Figure 2l,m).

### 2.2 Microtopography‐ and OM‐Induced Osteogenic Differentiation Depends on YAP and LATS1 Expression

**Figure 3:**
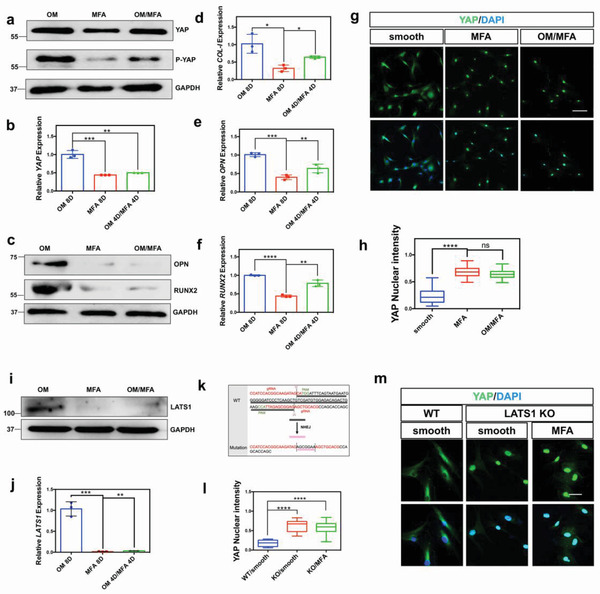
LATS1‐YAP switches on osteogenic signaling on the osteogenic medium (OM)/micro‐frustum‐array (MFA). a) The increase in total YAP protein expression was accompanied by increased phosphorylation on smooth substrates. The assessments were carried out on day 8. b) OM promoted YAP expression at the gene level. The data are presented as means ± SD, *p* values are based on Student's *t* test, *n* = 3, **p < 0.01, ***p < 0.001. c–f) After YAP deletion, both OM/MFA‐ and MFA‐induced osteogenesis was significantly weakened at the protein and gene levels. The data are presented as mean ± SD, *p* values are based on Student's *t* test, *n* = 3, *p < 0.05, **p < 0.01, ****p < 0.0001. g,h) MFA promotes the nuclear localization of YAP. After expanding on the substrate for 4 days, cells were fixed for immunofluorescence and analyzed using the anti‐YAP antibody. All data are presented as mean ± SD, *p* values are based on Student's *t* test, *n* > 3 per group, n.s, *p* > 0.05, ****p < 0.0001. Scale bar, 100 µm. i,j) MFA suppresses LATS1 expression. The data are presented as mean ± SD, *p* values are based on Student's *t* test, *n* = 3 per group, *p < 0.05, ****p < 0.0001. k) Genomic sequence of the CRISPR‐Cas9‐edited hMSCs clone that revealed on‐target deletion mutations in *LATS1*. The predicted gene alteration for each clone is indicated. l,m) After LATS1 KO, the nucleation rate of YAP on smooth substrates was significantly increased, similar to that on the MFA. All data are presented as mean ± SD, *p* values are based on Student's *t* test, *n* > 3 per group, ****p < 0.0001. Scale bar, 50 µm.

To explore the effect of YAP on hMSC regulation induced by OM and MFA, we designed YAP‐related positive and negative experiments. The dexamethasone in OM could promote the expression of YAP in cells.^[^ ^23^ ^]^ The expression of YAP is increased in response to dexamethasone in rat BMSCs and suggested that YAP is involved in the signaling pathway of dexamethasone with the help of LIM‐domain protein with 4.5 LIM domains (FHL2).^[^ ^23^ ^]^ Our experimental results indicated that OM promoted YAP expression (**Figure** **3**a,b) and that knockdown of YAP using small interfering RNA (siRNA) weakened the osteogenic efforts generated by the MFA or OM/MFA (Figure 3c–f; compared to Figure 1d–g), directly confirming that YAP plays an important role in MFA‐dependent osteogenesis. However, we found that on the smooth substrates, total YAP protein expression was increased, accompanied by an increase in phosphorylation, which was readily detected using a phospho‐YAP^S127^ antibody (Figures 3a and Figure S2a, Supporting Information), indicating that YAP protein was phosphorylated in the cytoplasm and did not enter the nucleus to act as a co‐transcription factor (Figure 3g,h). In contrast, the culture of competent hMSCs on MFA substrates tended to localize YAP in the cell nuclei compared to their culture on a smooth surface, with a decrease in phospho‐YAP^S127^ protein level (Figure 3g,h). Thus, MFA was found to promote YAP entry into the nucleus by inhibiting its phosphorylation.

To explore the mechanism underlying YAP phosphorylation and the effect of MFA, several possible genes were examined. Angiomotin family proteins (AMOTs) are important regulators of YAP expression and the cytoskeleton, and can directly bind to and potentially inhibit YAP.^[^ ^24^, ^25^ ^]^ Unfortunately, our results showed no significant differences in the expression of *AMOT* genes (Figure S2b, Supporting Information). Further, we detected several genes related to YAP phosphorylation, including Large Tumor Suppressor 1 (*LATS1*), Large Tumor Suppressor 2 (*LATS2*), and *14‐3‐3* (Figure S2c,d, Supporting Information; Figure 3i,j), of which *LATS1* was the most notable gene as changes in its expression were closely related to the effects of MFA. Our results also suggest that MFA significantly inhibits the expression of *LATS1* in hMSCs (Figure 3i,j), consistent with the previous experimental results suggesting that MFA inhibits YAP phosphorylation (Figure 2a,g). We also utilized CRISPR/Cas9 to engineer an hMSC line with *LATS1* knockout (KO). Genomic sequencing of CRISPR‐Cas9‐editing revealed an on‐target insertion mutation in the *LATS1* gene (Figure 3k). Notably, *LATS1* KO was sufficient to inhibit YAP phosphorylation and increase YAP nuclear translocation (Figure 3l,m). Therefore, it is reasonable to consider that the *LATS1* gene is crucial in the OM/MFA‐related signaling pathway.

### 2.3 Inhibition of LATS1 Contributes to the Osteogenic Differentiation of Competent hMSCs In Vitro and is Beneficial for Bone Formation and Repair In Vivo

To further verify the role of LATS1 in the osteogenic differentiation of hMSCs, we designed related experiments both in vitro and in vivo. Unexpectedly, reduced LATS1 expression without the induction by OM showed only a slight induction of osteogenic differentiation in hMSCs (Figure S3a, Supporting Information). However, when the hMSCs were subjected to osteogenic differentiation for 4 days in advance using the same method as described above (referred as competent hMSCs), the osteogenesis effect of *LATS1* KO became evident (Figure S3b,c, Supporting Information). These results were consistent with our previous results and verified the OM/MFA‐related signaling pathway.

**Figure 4:**
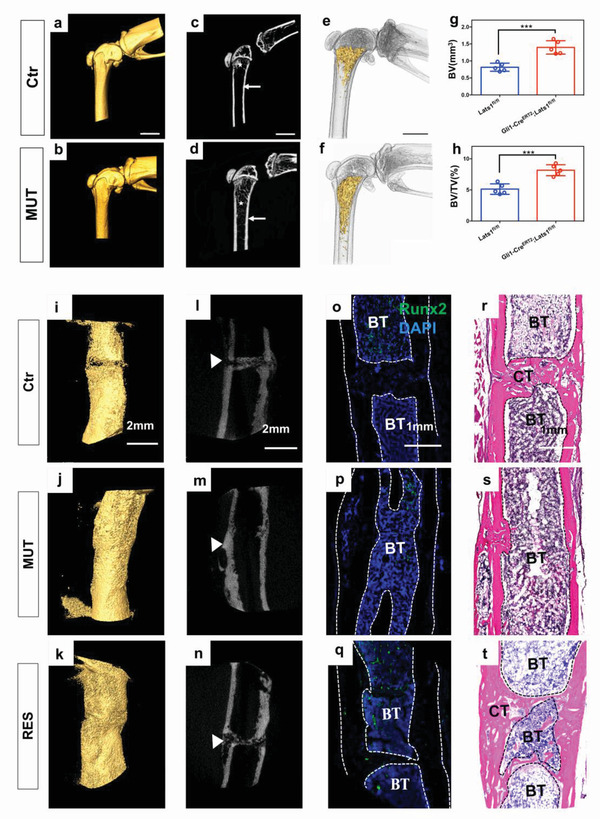
*Lats1* inhibition is beneficial for the development and repair process of bone, whereas inhibition of Rhoa, as a restored gene, prevents this process in vivo. a–f) MicroCT and digital 3D reconstruction of the femur in (a, c, e) Ctr (*Lats1* ^fl/fl^) mice and (b, d, f) MUT (Gli1‐Cre^ERT2^, *Lats1* ^fl/fl^) mice injected with tamoxifen ™ at 1 month of age and harvested at 2 months. Arrows and asterisks indicate the peripheral cortical bone and trabecular bone, which was increased in the *Lats1* KO group. Scale bar, 2 mm. g,h) Quantitative analyses showing the proportions of trabecular bone volumes. All data are presented as mean ± SD, *p* values are based on Student's *t* test, *n* = 5 per group, ***p < 0.001. i–k) Digital 3D reconstruction and l–n) microCT images of femur repair in Ctr, MUT, and RES (Gli1‐Cre^ERT2^, *Lats1* ^fl/fl^ with CCG‐1423 local injection) mice. Mice were induced with TM at 1 month of age, and were allowed to heal for 4 weeks before harvesting. Arrows indicate new bone formation in the defective area in the *Lats1* KO group. Rhoa inhibition slowed down the new bone formation induced by *Lats1*. Scale bar, 2 mm. o–q) Immunofluorescence of the femur fracture areas in mice at 4 weeks. Fracture healing of the MUT group was better than that of the Ctr and RES groups, indicating that *Lats1* inhibition is beneficial to bone repair whereas *Rhoa* inhibition weakens this process. Runx2 (green) activity confirmed the complete healing of MUT mice at 4 weeks. Scale bar, 1 mm. r–t) Images of HE staining results showing the healing status of fractured areas in mice at 4 weeks. Integrated fracture healing was observed in the MUT group. Scale bar, 1 mm. All data are presented as mean ± SD, *n* = 5 per group.

Next, we examined Lats1 protein expression in the long bone (femur) growth plate of 1‐month‐old *Lats1^fl/fl^* (Ctr), and *Gli1‐Cre^ERT2^; Lats1^fl/fl^* (MUT) mice. Gli1^+^ cells exist below the growth plate and are functionally important for supporting trabecular bone formation in postnatal mice.^[^ ^26^, ^27^ ^]^ We verified the role of *Lats1* in femur growth and the femur defect repair process (Figure S4, Supporting Information). The femur growth plate from the epiphysis to the diaphysis is divided into different zones, including reservation, proliferation, maturation and hypertrophy, and calcification and ossification zones. The immunofluorescence images showed that *Lats1* was mainly distributed in the reservation and proliferation zones, where osteogenesis is silent (Figure S5a–e, Supporting Information). In contrast, Yap and Runx2 were mainly distributed in the maturation and hypertrophy zones, and in the calcification zone, where osteogenesis is active. In the first set of experiments, we administered tamoxifen (TM) to the mice at 1 month after birth and harvested them at 2 months of age, which was the rapid growth period of the femur. In the *Lats1*‐null epiphyseal plate, we observed significantly increased bone mass including the trabecular bone and peripheral cortical bone, through computed tomography (CT) scanning and 3D reconstruction (**Figure** **4**a–f). Upon osteogenesis, in the zones of calcification and ossification, osteoclasts and osteoblasts from the diaphyseal side break down the calcified cartilage and replace it with mineralized bone tissue, respectively, forming trabecular bone.^[^ ^26^ ^]^ Our results showed that trabecular bone generation was more pronounced in the mutant group, indicating higher osteogenic activity (Figure 4g,h).

In another experiment, a 0.3‐mm‐wide bone fracture was created in the middle of the femur with the help of a bone position‐maintainer to determine whether *Lats1* KO contributed to bone repair. In subsequent experiments, we introduced the restored gene Ras homolog gene family member A (*Rhoa*), which is also proven to be involved in the OM/MFA signaling pathway as a *Lats1* antagonist in vitro. In the restored group (RES), *Gli1‐Cre^ERT2^; Lats1^fl/fl^* mice were injected with CCG‐1423 (Selleck, S7719), a specific *Rhoa* inhibitor.^[^ ^28^ ^]^ Consistent with normal femur growth, a corresponding increase in osteogenesis was observed when *Lats1* function was inhibited (Figure 4i–n; Figure S6a–f, Supporting Information). Especially at 4 weeks in the MUT group, the fracture disappeared, showing integrated healing. However, with *Rhoa* inhibition, the osteogenic benefit produced by *Lats1* was weakened, suggesting that *Rhoa* plays a contrasting role to that of *Lats1* in the osteogenic process. Immunofluorescence analysis of the fracture area showed that the fracture had completely healed in the MUT group with a decrease in osteogenic activity (Figure 4o–q). Although inhibition of *Rhoa* reduced the efficiency of osteogenesis, the RES group was still better than the control group, which might have been related to the inhibition of *Lats1* in the first month. Hematoxylin and eosin (HE) staining confirmed that the integrated healing in the MUT group and *Lats1* KO was beneficial for the repair of bone fractures (Figure 4r–t). In summary, we confirmed the unique effects of *Lats1* on osteogenesis in vitro and in vivo; however, the OM/MFA‐ specific signaling pathway remains to be supplemented with additional evidence.

### 2.4 OM/MFA Activates YAP via the INTA2‐FAK‐RHOA‐LATS1 Pathway

**Figure 5:**
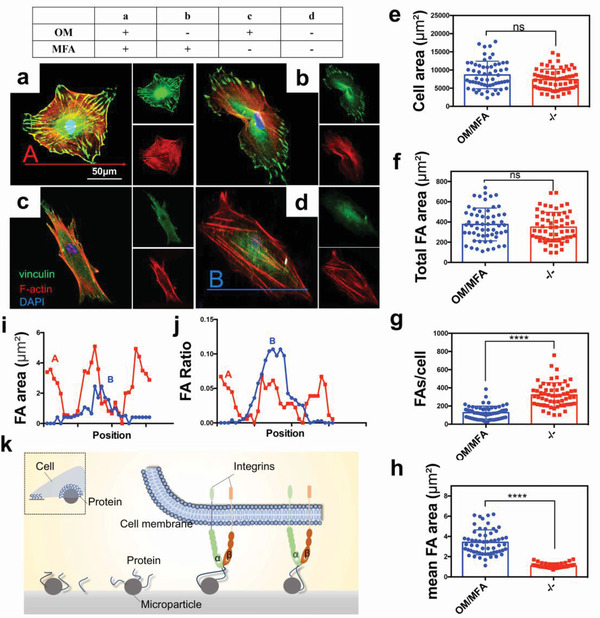
Increased numbers of focal adhesions mature on the micro‐frustum‐array (MFA) substrate. a–d) Immunofluorescence observations of cellular focal adhesions and cytoskeleton morphology using vinculin (green) and F‐actin (red) staining. OM represents hMSCs cultured in the osteogenic medium (OM) for 4 days. MFA represents hMSCs cultured on MFA substrates for 4 days. Scale bar, 50 µm. e–h) Corresponding quantitative analyses of cellular areas (e), total focal adhesion (FA) areas (f), average FA quantities (g), and mean FA areas (h). The data are presented as mean ± SD, *p* values are based on Student's *t* test, *n* = 59 per group, ns, *p* > 0.05, ****, *p* < 0.0001. i,j) Vinculin distribution in hMSCs as indicated by A and B in (a) and (d). k) Schematic representation of the MFA substrate in contact with the hMSC membrane. In the same projective area, microtopography provides more protein binding sites than the flat substrate, resulting in distinct sizes and spacing of the aggregated extracellular matrix (ECM) proteins on the microtopographical surface.

**Figure 6:**
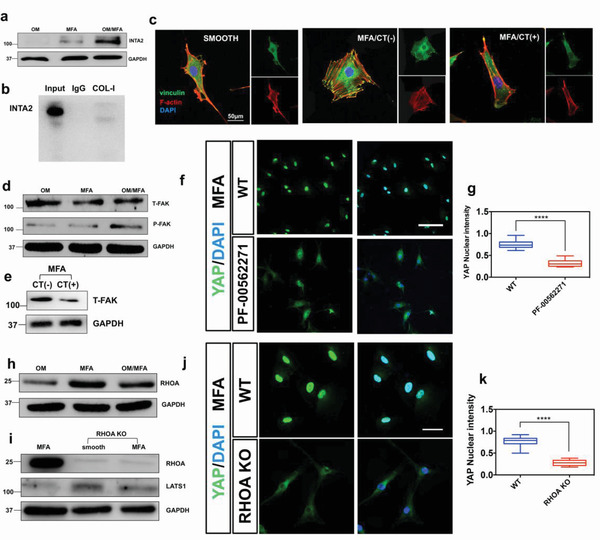
The micro‐frustum array (MFA) activates YAP via the INTA2‐FAK‐RHOA‐LATS1 pathway. a) INTA2 expression was consistent with that of COL‐I at the protein level. b) Co‐immunoprecipitation was conducted to verify the binding of COL‐I and INTA2. c) Immunofluorescence analysis of cellular focal adhesions and cytoskeletal morphology using vinculin (green) and F‐actin (red) staining. On the MFA, with integrin *α*_2_‐inhibiting CT, mature focal adhesions were decreased. Scale bar, 50 µm. d) Expression of total‐FAK and phosphorylated‐FAK. e) On the MFA, INTA2 inhibition decreased FAK expression. f) FAK inhibition reduced the role of microtopography in promoting the nuclear localization of YAP. Scale bar, 50 µm. g) Corresponding quantitative analyses of YAP nuclear localization. The data are presented as mean ± SD, *p* values are based on Student's *t* test, *n* >10 per group, ****p < 0.0001. h) MFA promoted RHOA expression. i) RHOA inhibition weakened the inhibitory effect of MFA on LATS1. j) RHOA inhibition reduced the role of microtopology in promoting YAP nuclear localization. Scale bar, 50 µm. k) Corresponding quantitative analyses of YAP nuclear localization. The data are presented as mean ± SD, *p* values are based on Student's *t* test, *n* >10 per group, ****p < 0.0001.

To better understand the OM/MFA‐specific signaling pathway, we further investigated the effects of OM and MFA on the cytoskeleton and morphology of hMSCs. First, we examined the morphology and the expression vinculin, a marker of focal adhesions in hMSCs (**Figure** **5**a–d; Figure S5, Supporting Information); the results showed no significant difference in the cellular areas between the MFA and smooth substrates (Figure 5e). Notably, however, on the MFA surface, hMSCs changed the original fusiform shape, became relatively polygonal, and showed a decreased aspect ratio, supporting that changing cell shape can, in turn, regulate their fate.^[^ ^29^ ^]^ The focus adhesion marker vinculin was found to be fewer but larger on MFAs than on the smooth substrates and tended to concentrate on cellular peripheries, suggesting the enhanced maturation of focal adhesions (Figure 5f–h and Figure 5i,j). Maturation of focal adhesions requires forces from their connections with actin filaments, as well as from the ensuing forces between integrins and the ECM.^[^ ^30^ ^]^ From a mechanical perspective, with the same projective area, the microparticles of MFA substrates provided more protein binding sites because of their spherical surface areas (Figure 5k). We also observed that OM promotes the formation of focal adhesions to some extent (Figure 5a–d). Ascorbic acid in OM was required as a cofactor for enzymes that hydroxylate proline and lysine in pro‐collagen, which could accelerate the secretion of COL‐I into the ECM.^[^ ^23^ ^]^ In the absence of ascorbic acid, proline could not be hydroxylated and collagen chains were not able to form a proper helical structure.^[^ ^23^ ^]^ COL‐I is considered a necessary protein for osteogenic differentiation, and hMSCs must be in contact with collagen‐containing ECM before they can undergo osteogenic differentiation.^[^ ^31^, ^32^, ^33^, ^34^ ^]^ Our results show that OM promotes the expression of COL‐I (Figure S1h,i, Supporting Information). Integrins, especially integrin *α*_2_, play an indispensable role in the sensory features and operation of connections in the ECM.^[^ ^35^, ^36^, ^37^ ^]^ Therefore, we evaluated integrin *α*_2_ of hMSCs on different substrates; the expression of integrin *α*_2_ was found to be consistent with that of COL‐I (**Figure** **6**a). Direct binding of COL‐I and integrin *α*_2_ was confirmed by protein co‐immunoprecipitation (Figure 6b). When integrin *α*_2_ was inhibited by its specific inhibitor cilengitide trifluoroacetate (CT, Selleck S7077), no significant difference in the number or volume of focal adhesions was observed between the MFA and smooth substrate groups (Figure 6c). Thus, we speculated that integrin *α*_2_ expression was induced by COL‐I as a receptor, and simultaneously as an operation device, it recruited the peripheral domains and a series of signaling modules to be further assembled into focal complexes or nascent adhesions. Focal adhesion kinase (FAK), a cytoplasmic tyrosine kinase, recruited by activated integrins along with other focal adhesion structural proteins are assembled into a focal adhesion complex.^[^ ^38^ ^]^ FAK in mature focal adhesions can translate mechanical signals into chemical signals and transmit them to cytoskeleton‐associated proteins.^[^ ^39^ ^]^ Our results at the gene and protein levels revealed that FAK expression in competent hMSCs on the MFA substrates was higher than that in the other groups (Figure 6d), consistent with the results of integrin and vinculin. FAK expression decreased significantly when integrin *α*_2_ was inhibited (Figure 6e). Further, PF‐00562271, a specific FAK inhibitor, significantly blocked FAK‐induced YAP nuclear localization (Figure 6f,g). These results provide compelling evidence that FAK participates in the OM/MFA pathway, thus promoting bone formation signaling.

The Rho GTPase family belongs to the small G protein superfamily, and more than 20 Rho family members have been discovered.^[^ ^40^ ^]^ According to their homology and functions, they can be divided into RHOA, CDC42, RAC1, and GTPase‐deficient groups.^[^ ^40^ ^]^ The Rho GTPase family are effectors of FAK signaling and are proven to be closely related to cytoskeleton formation.^[^ ^41^, ^42^ ^]^ We thus examined *RHOA*, *RAC1*, and *CDC42* gene expression in different substrates. Significantly, OM/MFA influenced *RHOA* expression but did not influence the expression of *RAC1* and *CDC42* (Figures 6h; Figure S7, Supporting Information). Later, we deleted *RHOA* using an siRNA, which impaired MFA‐induced LATS1‐inhibition (Figure 6i) and YAP nuclear localization (Figure 6j,k). These results indicate that RHOA serves as a connecting link between FAK and LATS1 in OM/MFA signaling. Overall, our results describe a new osteogenesis method that helps us understand the specific mechanism of OM and microtopography, synergistically promoting better osteogenesis (**Scheme** **1**).

### 2.5 HMFA‐Gel Biofilm Provides Chemical and Mechanical Cues to Cells and Enhances Bone Repair Efficiency In Vivo

**Figure 7:**
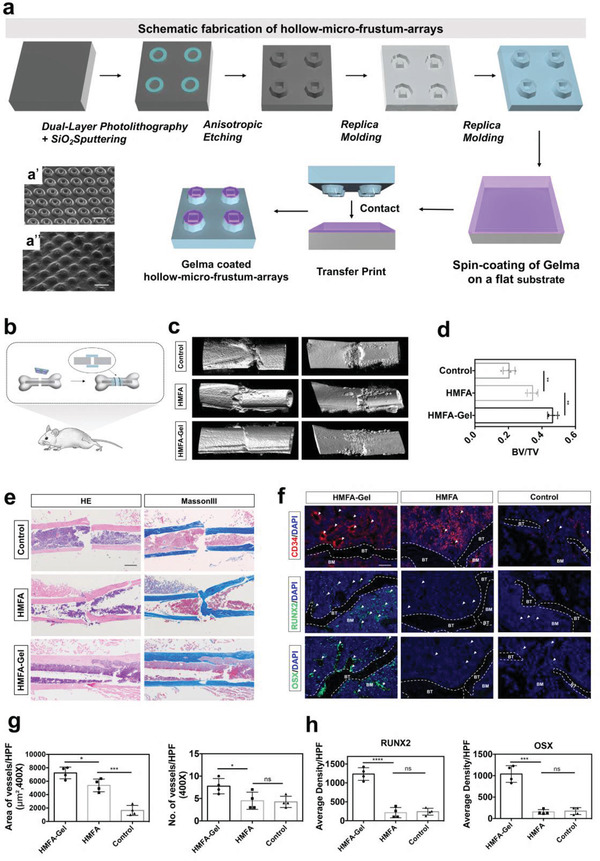
The hollow micro‐frustum‐array (HMFA)‐Gel membrane achieves better fracture healing. a) Fabrication process of the HMFA‐Gel. Scanning electron microscopic images of the HMFA (a’) and HMFA‐Gel (a’’) membrane. Scale bars, 5 µm. b) Schematic diagram of the HMFA‐Gel membrane fixed to the fracture surface. c) MicroCT images of femur repair at weeks 2 and 4. d) Quantitative analyses showing the proportions of trabecular bone volumes at 4 weeks. All data are presented as mean ± SD, *p* values are based on Student's *t* test, *n* = 4 per group, **p < 0.01. e) Images of HE staining and Masson‐III staining results showing the healing status of fractured areas in mice at 4 weeks. Integrated fracture healing was observed in the HMFA‐Gel group. Scale bar, 200 µm. f) Images of results of CD34, RUNX2, and OSX immunohistochemistry around the bone defect. BT = Bone tissue, BM = Bone marrow. Scale bar is 100 µm. g) Quantification of the area of vessels per high‐power field (HPF) (µm^2^) (×400, *n* = 4 per group) and number of vessels per HPF (×400, *p* values are based on Student's *t* test, *n* = 4 per group. ns, *p* > 0.05, *p < 0.05.***p < 0.001). h) Quantification of the density of RUNX2 and OSX expression per HPF (×400, *p* values are based on Student's *t* test, *n* = 4 per group. ns, *p* > 0.05, ****p < 0.0001).

To demonstrate that OM and microtopography synergistically promote better osteogenesis in vivo, we created a crater‐like pattern capable of loading OM with the help of methacrylated gelatin (GelMA) and called it the HMFA‐Gel membrane (**Figure** **7**a). The topography of the HMFA‐Gel membrane was similar to that of the MFA, while being able to release OM.^[^ ^43^ ^]^ The HMFA‐Gel membrane was then applied for fixation in the mouse femur bone fracture model (Figure 7b). Micro‐CT (Figure 7c) analysis showed complete bone healing at 4 weeks with the HMFA‐Gel compared to that with HMFA alone. The control group without any scaffold showed no significant signs of healing at the fracture end. Quantitative analyses showed that the proportions of trabecular bone volumes in the HMFA‐Gel group were significantly higher than those in the HMFA and control groups (Figure 7d). HE staining and Masson's trichrome staining showed that the HMFA‐Gel group showed better healing of the bone fracture (Figure 7e). Immunofluorescence analysis of the fracture area showed a higher percentage of CD34‐, RUNX2‐, and OSX (Osterix)‐positive cells in the HMFA‐Gel group than in the other two groups, indicating active angiogenesis and bone healing (Figure 7f–h).

**Figure 8:**
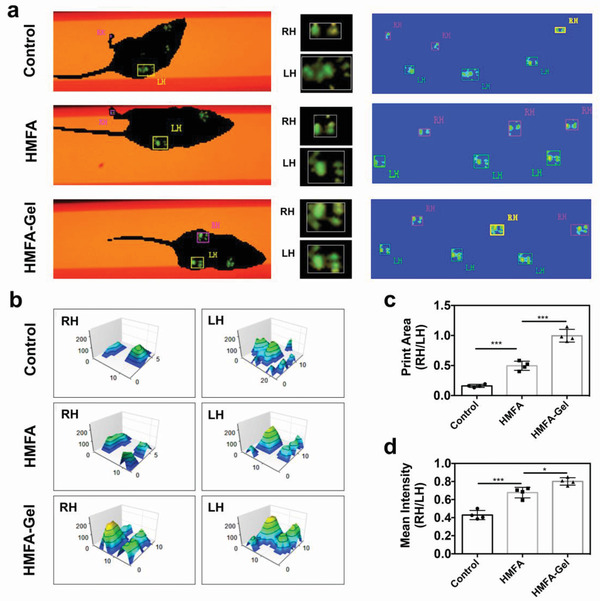
The HMFA‐Gel membrane helped restore the motor ability of mice. a) Representative images recording the footprints of the hind legs of mice during motion with the Catwalk XT at week 4. b) Measurement of 3D footprint intensities of mice at week 4. Horizontal changes in the print area (c) and mean intensity (d) among the groups at week 4. RH = right hind, LH = left hind. *p* values are based on Student's *t* test, *n* = 4 per group. *p < 0.05, ***p < 0.001.

Simultaneously, we evaluated the motor ability of mice using the CatWalk system. The footprints of the fracture hind limb (right hind) and the control hind limb (left hind) were captured (**Figure** **8**a) and converted to intensity for quantitative evaluation (Figure 8b). According to the print area, mice treated with HMFA‐Gel showed a better gait pattern (Figure 8c) with a mean intensity (Figure 8d) of the footprint close to 1, followed by that of mice in the HMFA and the control groups. These results suggest that the HMFA‐Gel membrane improved the walking performance of mice.

## 3 Discussion and Conclusions

The cell niche is a highly complex mechanical and chemical entity that regulates cellular behavior and tissue organization through a dynamic and bilateral dialogue between cellular sensing and interactive components. Currently, mechanical signals are increasingly being regarded as the primary regulators of cellular behavior, stemness maintenance, and tissue development and regeneration. Studies on micro‐topographical substrates have shown that they enhance the osteogenic differentiation of hMSCs compared to conventional tissue culture plates.^[^ ^44^, ^45^, ^46^ ^]^ Moreover, other studies have shown that microtopography induces neural, cartilage, or even adipogenic trans‐differentiation of hMSCs.^[^ ^47^, ^48^, ^49^, ^50^, ^51^ ^]^ Consistently, we found that osteogenic differentiation of hMSCs was inefficient when microtopography alone was used. However, if chemical factors were used to determine the osteogenic lineage of hMSCs in advance, the osteogenic induction effect of microtopography was greatly improved. Further, the benefits of synergy were far greater than a simple summation of the two, suggesting that OM enhances the mechanical sensitivity of cells.

Ascorbic acid is a cofactor of the enzyme that hydroxylates proline and lysine in pro‐collagen. The role of ascorbic acid in OM is mainly attributed to the secretion of collagen I into the ECM.^[^ ^23^ ^]^ Integrins (e.g., integrin *α*_2_) are activated by collagen I, which recruits peripheral domains, including signal modules, actin modules, and actin‐polymerizing modules that assemble into nascent adhesions.^[^ ^30^ ^]^ Based on the force of the natural contractility of hMSCs, nascent adhesions gradually shed or mature into focal adhesions depending on the anchorage between integrin and ECM.^[^ ^30^ ^]^ Adsorbing more proteins onto the surface along with distinct sizes and spacing of aggregated ECM proteins on the MFA induces changes in integrin‐mediated focal adhesion formation and stem cell function (Figure 6a–h). MFA promotes the formation of mature focal adhesions and activates the signal molecule *FAK*, thereby cascading a series of events. *FAK* can phosphorylate and activate guanine nucleotide exchange factors (GEFs) such as p190rhoGEF129, which may catalyze the exchange of GDP and GTP for the small rho family GTPases *RHOA*.^[^ ^52^, ^53^ ^]^ Moreover, dexamethasone in OM promotes the expression of *YAP* and *RUNX2*;^[^ ^34^ ^]^ however, YAP is limited to the cytoplasm. Activated *RHOA* facilitates the entry of *YAP* into the nucleus by inhibiting *LATS1* (Figure 6i–k). *YAP* directly binds to the transcription factor *RUNX2* in the nucleus and activates the expression of osteogenesis‐related genes.^[^ ^42^, ^54^ ^]^ Thus, OM and MFA signaling molecules cooperate to form a signaling path, which we termed OM/MFA signaling. Moreover, this suggests that the chemical and physical cues can synergistically regulate the behavior of cells.

To investigate the fitness between RHOA/LATS1 and MFA and to achieve precise gene therapy for bone regeneration, we designed relevant in vitro and in vivo experiments. The osteogenesis effect was not evident by the simple inhibition of LATS1 activity, similar to the in vitro results of the MFA group. We also determined and initiated the direction of hMSC differentiation, where the effect of LATS1 was significant on osteogenic differentiation in vitro. In both in vivo models, normal growth femur and bone defect femur in *Gli1‐Cre^ERT2^; Lats1^fl/fl^* mice, *Lats1* was knocked out in Gli1^+^ MSCs, which were more conductive to bone formation. We also introduced the *LATS1*‐antagonistic gene, *RHOA*, as a rescue gene. When *Lats1* was knocked out, *Rhoa* inhibition also slowed down the process of bone formation, indicating that *RHOA* and *LATS1* have a direct effect on osteogenesis. However, only microtopography was discussed in this work, additional research is needed to understand whether the INTA2/FAK/RHOA/LATS1/YAP signal axis is key in different mechanical signal transmission processes, such as hardness, polarity, and atomic structure.^[^ ^55^, ^56^, ^57^ ^]^

To realize the application of chemical and mechanical cues in combination in vivo, we created an HMFA‐Gel biofilm. HMFA was created using a new photolithography strategy called dual‐layer photolithography.^[^ ^58^ ^]^ Conventional UV photolithography is widely used for patterning micro‐and nanostructures for biological applications.^[^ ^59^, ^60^, ^61^ ^]^ However, the diffraction limit restricts the resolution of conventional photolithography to ≈0.5 to 1 micron. Dual‐layer photolithography utilizes the feature size difference between the two layers of photoresist to generate subtractive patterns. The gel‐hydrogel layer provided the biofilm with the ability to digitally regulate its 3D geometry, matching the specific macroscopic bone shape. And the HMFA‐Gel biofilm can wrap the bone defect model in a non‐invasive manner to avoid the contamination of invasive materials. In conclusion, dual‐layer photolithography provides a way to break the optical diffraction limit and realize sub‐micron patterning, and the biofilm can thus be extended to repair complex tissue defects.

Taken together, we propose a novel osteogenesis strategy that combines mechanical and chemical cellular factors and confirm that chemical cues induced mechanical sensitivity in the cells. And to the best of our knowledge, we also first reveal the pivotal role of INTA2/FAK/RHOA/LATS1/YAP in hMSC osteogenic differentiation both in vitro and in vivo. Finally, we fabricated a novel biofilm combined with chemical and mechanical cues to achieve cellular osteodifferentiation and to enhance bone repair efficiency in vivo.

## 4 Experimental Section

### Cultivation of hMSCs

hMSCs were extracted from the bone marrow aspirate (fluid) of healthy donors (aged 37–52 years) admitted to the First Affiliated Hospital of Medical College of Zhejiang University for iliac crest transplantation. The procedure was implemented with the approval of the local Scientific Ethics Committee (Research Ethics Committee of the First Affiliated Hospital, College of Medicine, Zhejiang University, approval number 20191556) and informed consent from all the donors. To obtain primary hMSCs, iliac crest marrow aspirates were subjected to density gradient centrifugation using the Histopaque‐1077 (Sigma‐Aldrich) lymphoid tissue separation reagent. Cells from the interlayer were seeded onto 100‐mm dishes (Corning), and hMSCs were selected using Dulbecco's modified Eagle's medium (DMEM, Gibco) supplemented with 10% fetal bovine serum (FBS, ScienCell). The cells were then cultured in a humidified atmosphere with 5% CO_2_ at 37 °C and later detached using 0.05% trypsin (Gibco) for further subculture; the cells were used for up to seven passages. The osteogenic medium (OM) consisted of growth media and osteogenic supplements with 10 nm dexamethasone and 0.2 mm ascorbic acid.

### Fabrication Process of PDMS‐Based Micro‐Frustum‐Arrays

Step 1: A Si (100) substrate with a thin layer of thermally grown silica (100 nm) was spin‐coated with a layer of negative photoresist SUN 9i (Suntific Materials Inc., China). Photolithography was applied to generate microdisk arrays of photoresist (diameter: 5 µm; pitch: 10 µm). Reactive ion etching (RIE‐280, Hefei Jusheng Vacuum Technology Inc.) was applied to transfer the micro‐disk‐array pattern to silica, followed by removal of the photoresist. Step 2: The prepared silica micro‐disk arrays were used as a resist in the subsequent anisotropic etching of Si (100) (in 30% KOH aqueous solution) to generate Si micro‐frustum arrays. Step 3: Heat‐assisted imprinting was used to replicate the negative pattern of the micro‐frustum arrays onto a PMMA substrate. Step 4: A second replica molding process was used to generate PDMS‐based micro‐frustum arrays.

### Fabrication Process of PDMS‐Based Hollow‐Micro‐Frustum‐Arrays

Step 1: The recently developed dual‐layer photolithography was used to pattern hollow‐micro‐ring‐arrays on the dual‐layer photoresist, as reported previously.^[^ ^58^ ^]^ Next, a thin‐layer of silica (60 nm in thickness) was deposited on top via magnetron sputtering (DM‐300, Hefei Jusheng Vacuum Technology Inc.); after a lift‐off process, silica micro‐ring‐arrays were fabricated on the Si substrate; Step 2: Silica micro‐ring‐arrays were used as the resist in the anisotropic etching of Si (100) (30% KOH solution) to generate Si hollow‐micro‐frustum‐arrays. Step 3: Heat‐assisted imprinting was used to replicate the negative pattern of hollow‐micro‐frustum arrays on a PMMA substrate. Step 4: A second replica molding process was used to generate the PDMS‐based hollow‐micro‐frustum arrays. The surface microdot topology was characterized using a scanning electron microscope (SEM, SU‐70Hitachi). The roughness of the MFA was measured using a confocal laser scanning microscope (LSM700, Zeiss).

### Fourier Transform Infrared (FTIR) Spectroscopy Analysis

A FTIR spectrophotometer (Spectrum 400, PerkinElmer, MA, USA) was used to collect infrared spectra of the PDMS and PDMS‐MFA. Infrared spectra were collected between 4000–400 cm^−1^ at a resolution of 4 cm^−1^ using 32 scans.

### Atomic Force Microscopy Analysis

Images (field size: 50 × 50 µm) of the substrate were collected by AFM, which was performed in contact mode with a scan rate of 1.0 Hz. The acquired images were analyzed using NanoScope Analysis version 1.90 to reconstruct digital 3D structure.

### Substrate Contact Angle Analysis

A 4‐µL drop of distilled water was dropped onto the surface of the substrate, and the contact angle was analyzed by the sessile drop technique (OCA15plus, DataPhysics Instruments GmbH, Germany). The contact angle of each drop was recorded at equilibrium. The right and left contact angles were averaged to obtain a mean *θ* value.

### Immunofluorescence Staining

The hMSCs cultured on the substrates were fixed using 4% paraformaldehyde for 15 min and then permeabilized with 0.05% Triton X‐100 (Sigma‐Aldrich) for 10 min at room temperature. After blocking the slides with 2% fetal bovine serum (FBS, Sigma‐Aldrich) and 2% bovine serum albumin (BSA, Sigma‐Aldrich) in phosphate‐buffered saline (PBS, Sigma‐Aldrich) for 1 h, primary antibodies (Table S1, Supporting Information) were added and incubated at 4 °C overnight. Alexa Fluor‐488 conjugated anti‐mouse IgG secondary antibody (Invitrogen, 1:200) was used as the secondary antibody. The fluorescent dye rhodamine‐phalloidin (PHDR1 Cytoskeleton, 1:200) and 4′,6‐diamidino‐2‐phenylindole (DAPI, H‐1200 VECTOR,1:1000) were used to observe the cytoskeleton and nuclei, respectively. Confocal laser scanning microscopy (CLSM, Nikon A1R/A1) was used to observe the corresponding protein locations.

For alkaline phosphatase (ALP) staining, hMSCs were fixed in 4% paraformaldehyde for 20 min and stained using the BCIP/NBT alkaline phosphatase color development kit (Beyotime) for 30 min at room temperature, avoiding natural light. For Alizarin Red S staining, hMSCs were fixed in 4% paraformaldehyde for 20 min and then incubated in 3‐mm ARS (Sigma‐Aldrich) for 30 min at room temperature.

### CRISPR‐Cas9 Gene Editing

The *LATS1* KO cell line used in this study was generated using the CRISPR‐Cas9 gene‐editing approach. The guide RNA for *LATS1* sequences was as follows: *LATS1*‐guide A: CGTGCAGCTCTCCGCTCTAA and *LATS1*‐guide B: CCATCCACGGCAAGATAGCA. The gRNA sequences were cloned into the plasmids pSpCas9(BB)‐2A‐Puro (PX459) or PX330‐U6‐Chimeric_BB‐CBh‐hSpCas9 (gifted by Dr. Feng Zhang). The restructured plasmids were transiently transfected into hMSCs. After 24 h of transfection, the cells were cultured in puromycin (2 µg mL^−1^) for 48 h. The individual KO colonies were validated by western blotting and gene sequencing.

### Real‐Time Quantitative Reverse Transcription Polymerase Chain Reaction (RT‐PCR)

RT‐PCR was used to detect the expression of osteogenesis‐related and substrate‐specific genes. Total RNA was extracted from hMSCs that were seeded on the substrates using TRIzol reagent (Invitrogen). cDNA synthesis was performed using the Superscript II first‐strand cDNA synthesis kit (TaKaRa), and qPCR reaction performed on an ABI ViiA7 Sequence Detection System (Applied Biosystems) using the SYBR Premix Ex Taq kit (TaKaRa). Target gene expression levels were analyzed using SDS 2.1 software (Applied Biosystems) with the 2^−ΔΔCT^ method and were normalized using the housekeeping gene, GAPDH. A detailed list of the primer sequences used here is provided in Table S2, Supporting Information.

### Western Blot Analysis

The total proteins of hMSCs were extracted using RIPA buffer (Thermo Scientific) with protease and phosphatase inhibitors (DAWEN BIOTECH), separated by 10% sodium dodecyl sulfate (SDS)‐polyacrylamide gel electrophoresis, and transferred onto a polyvinylidene fluoride membrane (Millipore). The membrane was then blocked using 5% non‐fat dry milk dissolved in TBST for 2 h at room temperature with gentle shaking and further incubated with primary antibodies (Table S1, Supporting Information) at 4 °C overnight, followed by addition of the corresponding horseradish peroxidase‐conjugated secondary antibodies. Protein expression was detected using the Bio‐Rad ChemiDoc Touch system (Bio‐Rad) and was quantitated using ImageJ software (National Institutes of Health).

### Co‐Immunoprecipitation Assay

The total proteins of hMSCs were extracted using RIPA solution (Thermo Scientific) with protease and phosphatase inhibitors (Dawen Biotech). Human IgG and primary antibodies were added at a ratio of 2 µg mg^−1^ total protein, and the mixtures were then incubated at 4 °C for 1 h. Protein A/G agarose (Santa Cruz Biotechnology) was then added at 30 µL per sample, and the mixtures were incubated at 4 °C overnight. Lysis buffer was used for washing the solution five times, and the protein mixtures were denatured using western loading buffer without SDS before immunoblotting analysis.

### Transfection of hMSCs with siRNA

The siRNA duplexes were constructed by Shanghai GenePharma Co., China (RHOA 5′‐GAAGGAUCUUCGGAAUGAUTT‐3′.

5′‐AUCAUUCCGAAGAUCCUUCTT‐3′; YAP
5′‐CUGCCACCAAGCUAGAUAATT‐3′
5′‐UUAUCUAGCUUGGUGGCAGTT‐3′; MOCK
5′‐UUCUCCGAACGUGUCACGUTT‐3′ and 5′ACGUGACACGUUCGGAGAATT‐3′).

hMSCs were collected in a suspension by detaching using 0.05% trypsin (Gibco) and were then transfected with the corresponding siRNA or negative control (mock) using Lipofectamine 3000 (Invitrogen).

### Animals

*Gli1‐Cre^ERT2^* (JAX no. 0 07913) and *Lats1^fl/fl^* (JAX no. 02 4941) mouse strains were used in this study. The mouse experiments were approved by the Institutional Animal Care and Use Committee of Zhejiang University (approval number X1503142) and were performed according to the set regulations for animal experiments. All mice were housed under a 12 h light/dark cycle under pathogen‐free conditions with free access to food and water. All mice were identified using ear tags and PCR‐based genotyping. The animals were euthanized by an overdose of carbon dioxide and were then decapitated for sample acquisition. One‐month‐old *Gli1‐Cre^ERT2^; Lats1^fl/fl^* mice were induced with tamoxifen (TM) (Sigma‐Aldrich), and the femurs were then collected or prepared for surgery at 1‐month post‐induction; age‐matched *Lats1^fl/fl^* mice were used as controls. TM was mixed in corn oil (Sigma‐Aldrich) at 20 mg mL^−1^ and 1.5 mg per 10 g of the body weight of each mouse and injected intraperitoneally (i.p.) for 2 consecutive days.

The mouse femur was fractured as previously described.^[^ ^62^ ^]^ Briefly, the animals were kept in a ventral recumbent position with the left hind limb extension. A standard approach was used for aseptic surgery to expose the anterolateral surface of the femur. The skin, fascia lata, and muscles were carefully incised to expose the full length of the femur, preserving the sciatic nerve caudally, and the articular capsule distally. A peek microlocking plate (10‐mm‐long and 1.5‐mm‐wide; MouseFix Plate; RISystem) with self‐tapping lock screws (2‐mm‐long; MouseFix Screw; RISystem) was used to fix the femur. A Saw Guider (MouseFix Drill‐&Saw guide single cut; RISystem) and Gigli saws (0.22 mm; RISystem) were then used to produce a femoral defect. Later, the fascia and subcutaneous planes were closed using a 5.0 glycomer, and skin closure was accomplished using a 4.0 glycomer. Analgesia was provided to all mice through subcutaneous administration of buprenorphine (0.1 mg per kg animal weight) every 12 h post‐surgery for 3 consecutive days. For LATS1 restored groups, CCG‐1423 (Selleck, S7719), a specific RHOA pathway inhibitor, was injected locally every other day at a concentration of 1 µm until the samples were harvested.

Animal experiments using HMFA‐Gel membrane: The mice were anesthetized by 1% pentobarbitone (0.14 µL g^−1^). Then, the mice were kept in a ventral recumbent position with the left hind limb extension. A standard approach was used for aseptic surgery to expose the anterolateral surface of the femur. The skin, fascia lata, and muscles were carefully incised to expose the full length of the femur, preserving the sciatic nerve caudally, and the articular capsule distally. A Saw Guider (MouseFix Drill‐&Saw guide single cut; RISystem) and Gigli saws (0.22 mm; RISystem) were then used to produce a femoral defect. The femur was sawed at a width of 0.3mm. The intramedullary nail was used for the initial fixation of sawn bones. As for the experiment group, the fabricated HMFA‐Gel membrane was wrapped around the fracture. Then, the membrane, the fascia and subcutaneous planes were closed using a 5.0 glycomer, and skin closure was performed using a 4.0 glycomer. Analgesia was provided to all mice through subcutaneous administration of buprenorphine (0.1 mg per kg animal weight) every 12 h post‐surgery for 3 consecutive days. 4 weeks for healing before harvesting were allowed. The HMFA‐Gel membrane did not degrade when the sample was harvested and the HMFA‐Gel membrane was removed during the femur histological examination.

### MicroCT Imaging

The femur bones were radiographed in live mice using a microCT scanner (Scanco Microct µ100) with a resolution of 20 µm. Images were collected using a 70‐KVp and 114‐µA X‐ray source. All 3D reconstructions and sections were analyzed using ImageJ software.

### HE and Immunofluorescence Staining

Femur samples were fixed overnight at 4 °C using 4% paraformaldehyde in PBS (Sigma‐Aldrich), followed by decalcification in ethylenediaminetetraacetic acid (Sigma‐Aldrich) for ≈2 weeks. Dehydration was performed using a graded sucrose solution (incubated in 15% and 30% sucrose for 2 h each at room temperature and then in 30% sucrose with 50% Optimum Cutting Temperature compound (OCT, Sakura Finetek) overnight at 4 °C), followed by immediate embedding in OCT. The frozen tissue blocks were sectioned at 8 µm thickness on a cryostat (Leica) and mounted on SuperFrost Plus slides (Fisher) for staining.

For immunostaining, sections were permeabilized using blocking buffer containing 1% BSA, 2% goat serum, and 0.3% TritonX‐100 in PBS for 1 h at room temperature and then incubated overnight at 4 °C with the primary antibodies (Table S2, Supporting Information). The next day, sections were incubated with Alexa Fluor 488 goat anti‐rabbit IgG (Invitrogen, 1:150) along with DAPI for 1 h at room temperature and mounted using Vectashield mounting medium (H‐1000, Vector Laboratories). Images were captured using CLSM.

### Statistical Analyses

All data are expressed as the mean ± standard deviation. All experiments were conducted with a minimum of *N* = 3 for each data point. One‐way analysis of variance or the Student's *t*‐test was used for statistical analyses, which were performed using Prism and SPSS Statistics software. Statistical significance was set at *p* < 0.05. The error bars in all figures indicated the standard errors.

## Conflict of Interest

The authors declare no conflict of interest.

## Author Contributions

J.H, D.Y., and Q.L. contributed equally to this work. J.H., D.Y., Q.L.: Conception and design, collection and/or assembly of data, data analysis and interpretation, manuscript writing; S.D., X.H.: Provision of study material and data analysis; collection and/or assembly of data; H.Z., X.W., C.L., X.Y.: Data analysis and interpretation, manuscript revision/editing; J.W., H.K., Z.J., Xz.X: Nanofabrication, SEM, and AFM. All authors have read and approved the final manuscript. M.Y., H.W., Xb.X: Conception and design, financial support, administrative support, manuscript revision/editing, final approval of manuscript.

## Supporting information

Supporting Information

## Data Availability

The data that support the findings of this study are available from the corresponding author upon reasonable request.
