# Supplementary material for: Osteogenesis‐Inducing Chemical Cues Enhance the Mechanosensitivity of Human Mesenchymal Stem Cells for Osteogenic Differentiation on a Microtopographically Patterned Surface
Source: Adv Sci (Weinh). 2022 Apr 4;9(16):2200053. doi: 10.1002/advs.202200053 (PMC9165486; doi:10.1002/advs.202200053)
Supplement: Supplementary file 1 — Supporting Information [file ADVS-9-2200053-s001.pdf]

ADVANCED SCIENCE
Open Access

Supporting Information

for *Adv. Sci.*, DOI 10.1002/advs.202200053

Osteogenesis-Inducing Chemical Cues Enhance the Mechanosensitivity of Human Mesenchymal Stem Cells for Osteogenic Differentiation on a Microtopographically Patterned Surface

Jianxiang He, Dongqi You, Qi Li, Jiabao Wang, Sijia Ding, Xiaotong He, Haiyan Zheng, Zhenkai Ji, Xia Wang, Xin Ye, Chao Liu, Hanyue Kang, Xiuzhen Xu, Xiaobin Xu, Huiming Wang* and Mengfei Yu**

Osteogenesis-inducing chemical cues enhance the mechanosensitivity of human mesenchymal stem cells for osteogenic differentiation on a microtopographically patterned surface

Jianxiang He, Dongqi You, Qi Li, Jiabao Wang, Sijia Ding, Xiaotong He, Haiyan Zheng, Zhenkai Ji, Xia Wang, Xin Ye, Chao Liu, Hanyue Kang, Xiuzhen Xu, Xiaobin Xu, Huiming Wang & Mengfei Yu

SUPPLEMENTARY MATERIALS

Supplementary Figures and Legends

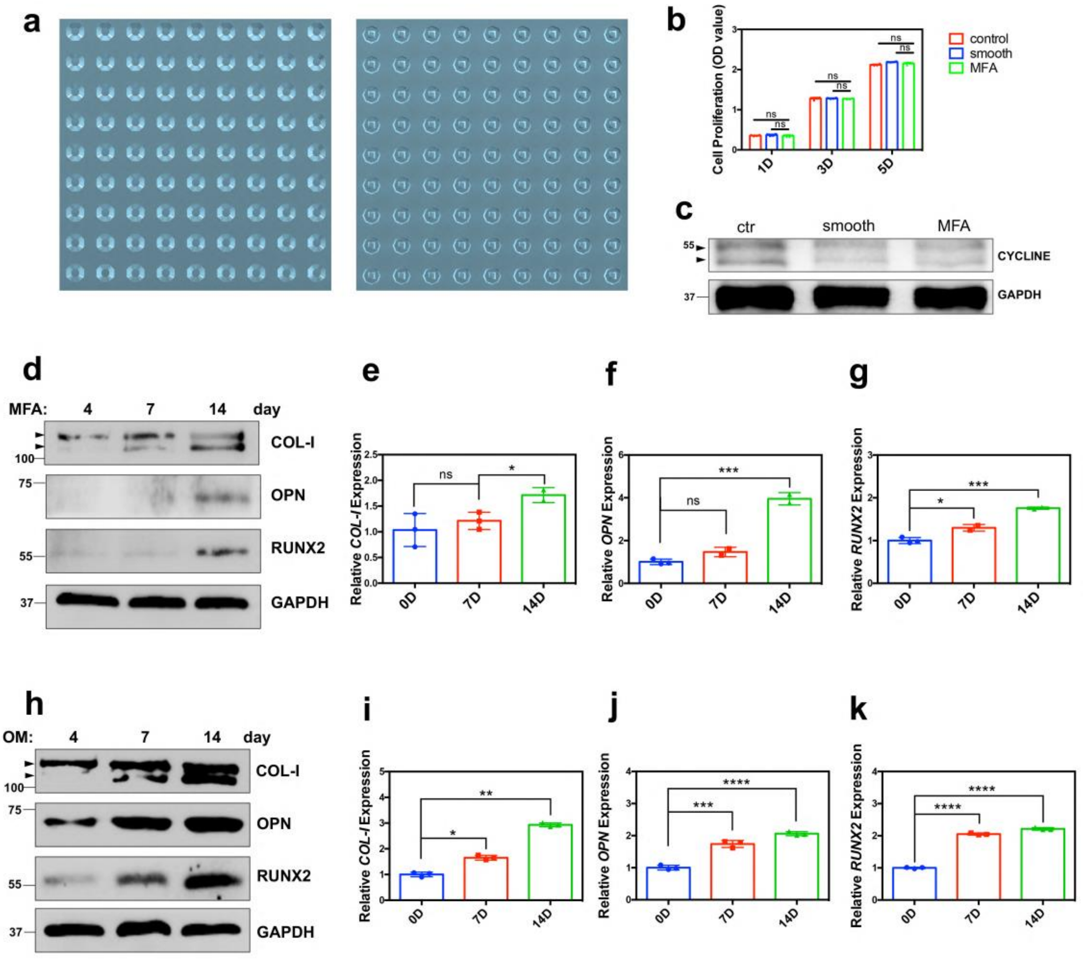

Figure S1. Biocompatibility of the micro-frustum-array (MFA) and the effects of MFA and osteogenic medium (OM) on hMSC osteogenesis. (a) Schematic diagram showing the topology of MFA and HMFA. **(b)** hMSCs proliferation (1, 3, and 5 days) on cell culture dishes, smooth substrates, and MFA. The data are presented as mean ± SD; p values are based on Student's t test, n = 3 per group. ns, $p > 0.05$. **(c)** Western

blot analyses of the proliferation protein CYCLIN E in hMSCs cultured on cell culture dishes, smooth substrates, and MFA (24 h). **(d, h)** Western blot analyses of the relative protein expression of osteogenic differentiation markers COL-I, OPN, and RUNX2 in hMSCs cultured on **(d)** MFA substrates and **(h)** in OM on days 4, 7, and 14. **(e-g, i-k)** qPCR analyses of osteogenic gene expression in hMSCs on **(e-g)** MFA substrates and **(i-k)** in OM on days 4, 7, and 14. The data are presented as mean ± SD; p values are based on Student's t test, n = 3 per group. ns, $p > 0.05$, *, $p < 0.05$; **, $p < 0.01$; ***, $p < 0.001$; ****, $p < 0.0001$.

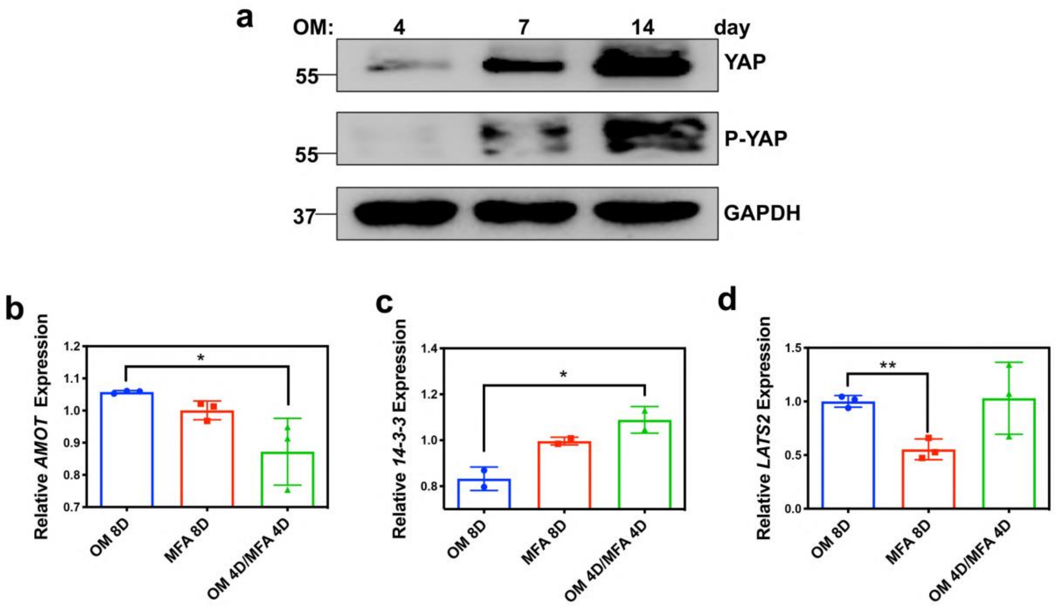

Figure S2. YAP-related inhibition of the gene expression. (a) Western blot analyses of relative YAP and P-YAP protein expression in hMSCs cultured in OM for 4, 7, and 14 days. **(b-d)** qPCR of *AMOT*, *14–3–3*, and *LATS2* gene expression under

different conditions. OM represents hMSCs cultured in the osteogenic medium (OM) for 8 days; MFA represents hMSCs cultured on the MFA substrate for 8 days; OM/MFA represents hMSCs cultured in OM for 4 days and then cultured on the MFA substrates for another 4 days. The data are presented as mean ± SD; p values are based on Student's t test, n = 3 per group. Ns, $p > 0.05$; *, $p < 0.05$; **, $p < 0.01$.

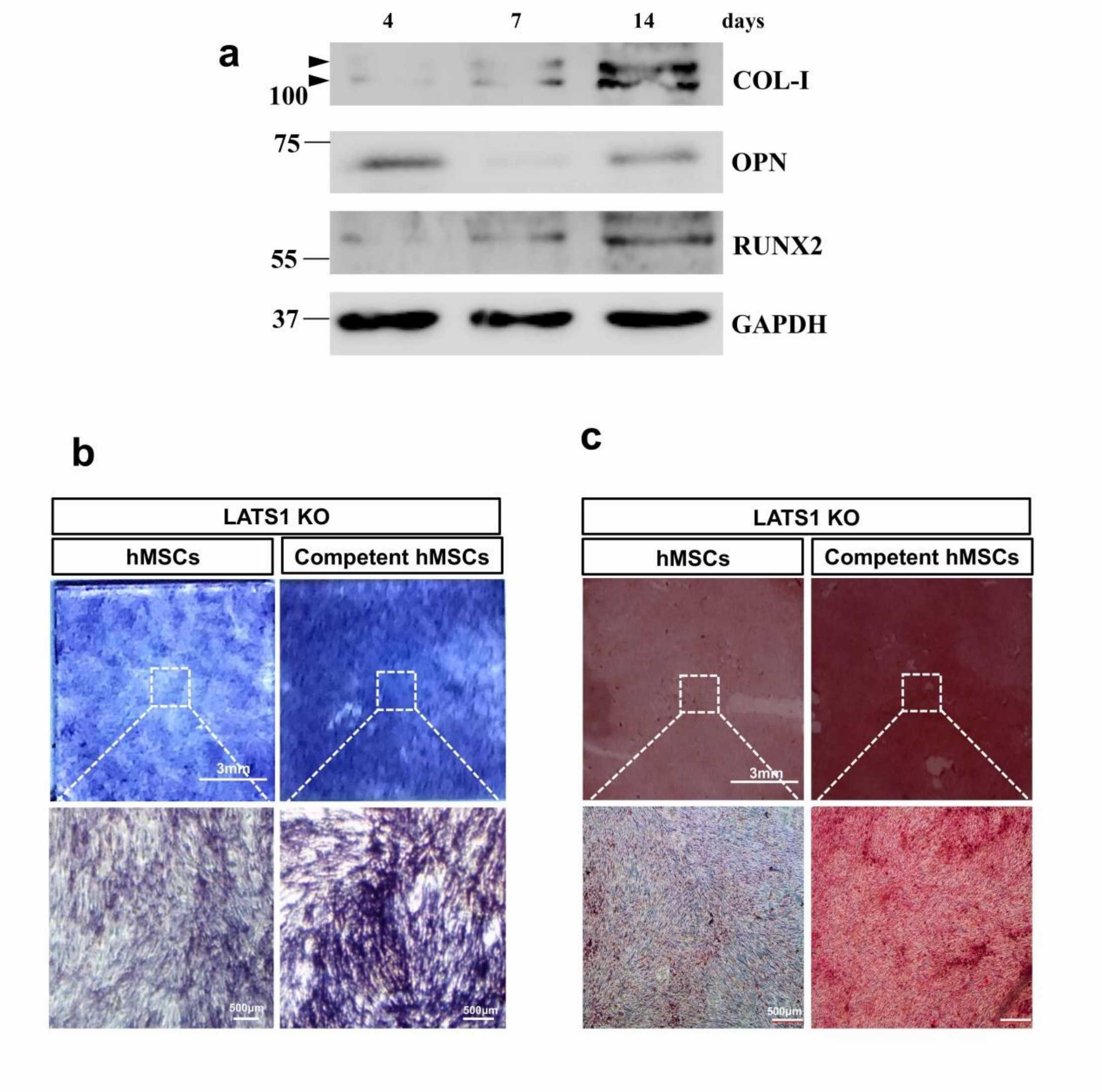

Figure S3. Effect of LATS1 on hMSCs osteogenic differentiation *in vitro.* (a) Western blot analyses of osteogenic proteins in LATS1 KO hMSCs. **(b)** ALP and **(c)** ARS activity analyses of osteogenic proteins in LATS1 KO hMSCs and in competent hMSCs. Scale bars, top 3 mm, bottom 500 μm.

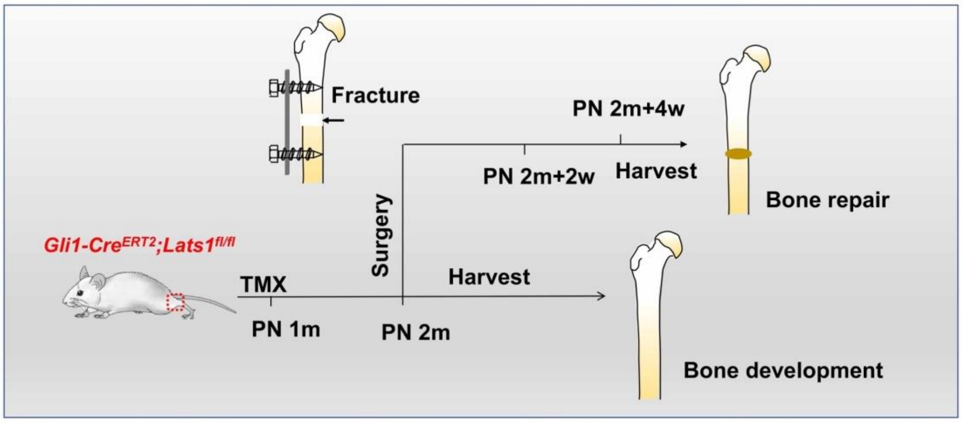

Figure S4. Schematic diagram showing the experimental process identifying the relationship between target genes in the development and repair of the femur in mice.

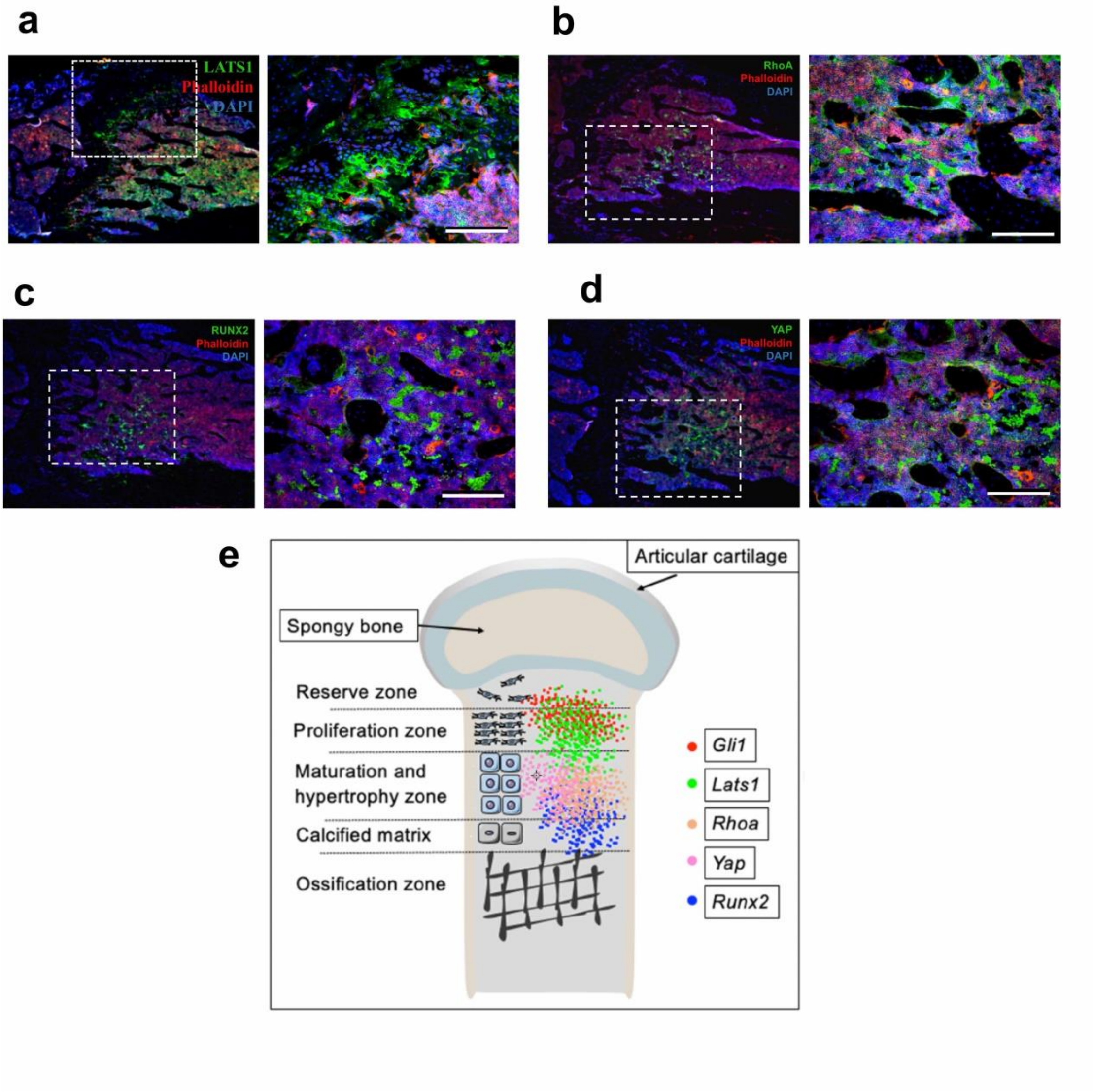

Figure S5. The different zones of femoral growth plate. (a-d) Immunofluorescence results showing the distributions of relevant genes. *Lats1* was mainly concentrated in the reserve and proliferation zone, where osteogenesis is silent. *Yap*, *Rhoa*, and *Runx2* were mainly concentrated in the maturation and hypertrophy zone as well as in the calcification and ossification zones, where osteogenesis is active. Scale bars, 2 mm. **(e)** Schematic diagram showing the structure of the femur epiphyseal plate and the distribution of related genes.

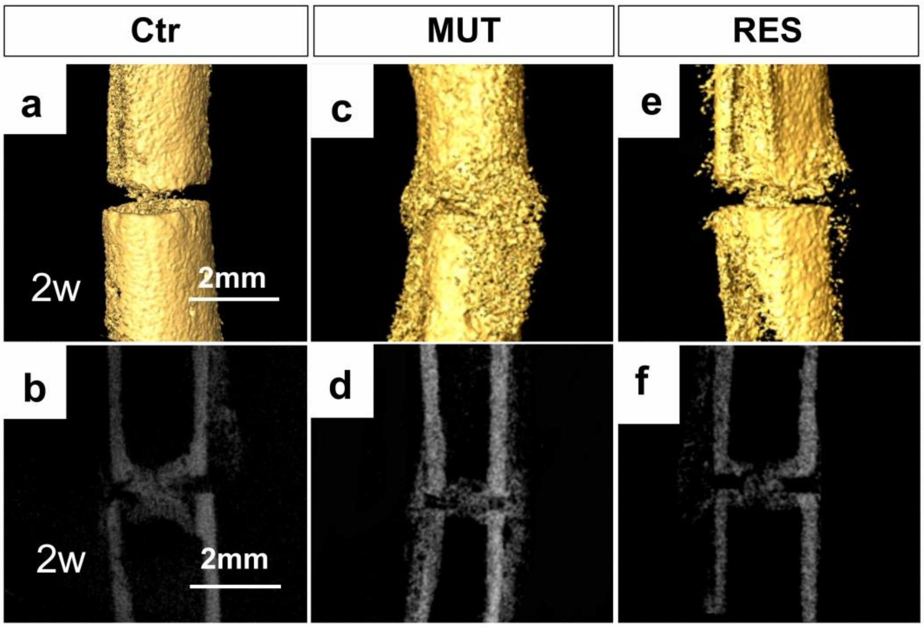

Figure S6. Inhibition of *Lats1* is beneficial to bone development *in vivo.* (a, c, e) Digital 3D reconstruction and **(b, d, f)** microCT images of femur repair in Ctr mice, MUT mice, and RES (Gli1-Cre^{ERT2}, $\textit{Lats1}^{fl/fl}$ with CCG-1423 local injection) mice. The mice were induced with tamoxifen ™ at 1 month of age, and before harvesting, they were allowed to heal for 2 weeks. New bone formation was observed in the defective area in the *Lats1* KO group, whereas the inhibition of *Rhoa* slowed the new bone formation induced by *Lats1*. Scale bar, 2 mm. n = 5 per group.

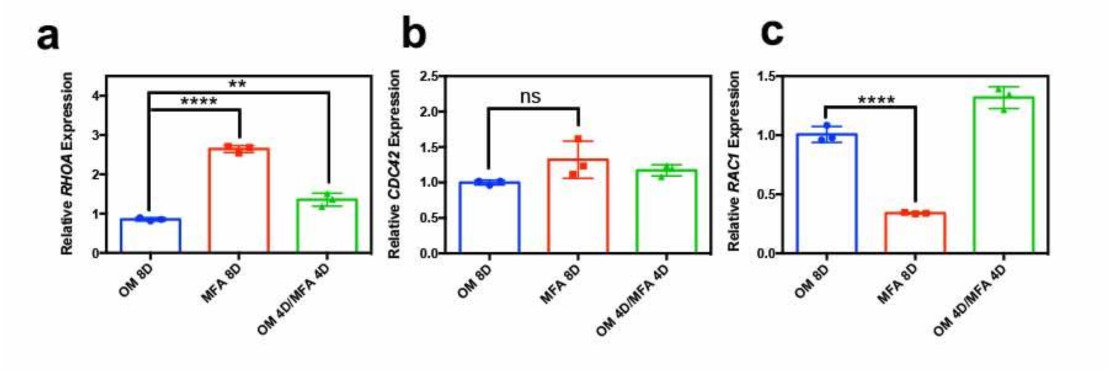

Figure S7. The influence of OM/MFA on RHOA, RAC1, and CDC42. (a-c) qPCR of *RHOA*, *RAC1*, and *CDC42* at different conditions. OM/MFA influenced the expression of *RHOA* but did not influence *RAC1* and *CDC42*. The data are presented as mean ± SD; p values are based on Student's t test, n = 3 per group. ns, p > 0.05; **, p < 0.01; ***, p < 0.001.

ID	Number	Application
COL-I	Abcam, ab34710	WB 1:1000
OPN	Abcam, ab8448	WB 1:1000
RUNX2	Abcam, ab236639	WB 1:1000 IHF 1:100
RHOA	Abcam, ab54835	WB 1:2000 IHF 1:200
LATS1	Santa Cruz Biotechnology, sc-398560	WB 1:1000 IHF 1:200

YAP	Santa Cruz Biotechnology, sc-271134	WB 1:1000 IHF 1:200 ICF 1:100
P-YAP	Abcam, ab76252	WB 1:1000
FAK	Abcam, ab40794	WB 1:1000
P-FAK	Abcam, ab91298	WB 1:1000
INTA2	Abcam, ab133557	WB 1:1000
Vinculin	Sigma-Aldrich, V9131	ICF 1:200
CyclinE	Abcam, ab33911	WB 1:1000
GAPDH	Abcam, ab9485	WB 1:5000

Table S1. Primary antibodies used for western blot (WB) and immunofluorescence (IHF, ICF) in this study

Gene	Primer Sequences (5′-3′) F	Primer Sequences (5′-3′) R
COL-I	CCAAAGGATCTCCTGGTGAA	GGAAACCTCTCTCGCCTCTT
OPN	AGCAGCTTTACAACAAATACCCAG	TTACTTGGAAGGGTCTGTGGG
RUNX2	AGATGATGACACTGCCACCTCTG	GGGATGAAATGCTTGGGAACT
RHOA	GAGCCGGTGAAACCTGAAGA	CCCCAGAGCTATGCCAACAA
RAC1	AAACCGGTGAATCTGGGCTT	AGAACACATCTGTTTGCGGA

CDC42	AACGCCCCGGTGGAGAA	ACACGAGTGCATGTGGGTAG
AMOT	GGCCACTTCCTAACCAGCAT	ACGCTCTCTCAGATCCCTGT
14--3--3	CACGGTGCTGGAATTGTTGG	TCGATCATCACCACACGCAA
LATS2	AATGTCCCACTTGGGTCTGC	CCGAAGACTTGGATGGCTGT
LATS1	GCTGCACCAAAACCCATCTG	TTTAAAAGGTTTTACCCGCATCA
YAP	CCCTCGTTTTGCCATGAACC	GTTGCTGCTGGTTGGAGTTG
GAPDH	CGTCTTCACCACCATGGAGA	CGGCCATCACGCCAGTTT

Table S2. Primer sequences used in the study
